# Epithelioid Sarcoma of the Forearm in a 70‐Year‐Old Woman: A Case Report at a Referral Facility in Tanzania

**DOI:** 10.1002/ccr3.72171

**Published:** 2026-03-06

**Authors:** Furaha E. Kasyupa, Jeremia J. Pyuza, Abitalis Mayengela, Agumbwike Mwakitwange, Deardiana Sengelela, Gilbert Nkya, Patrick Amsi, Angela Pallangyo, Alex Mremi

**Affiliations:** ^1^ Department of Pathology KCMC University Moshi Tanzania; ^2^ Department of Pathology Kilimanjaro Christian Medical Centre (KCMC) Moshi Tanzania; ^3^ Kilimanjaro Clinical Research Institute Moshi Tanzania

**Keywords:** distal‐type, elderly patient, epithelioid sarcoma, soft tissue sarcoma

## Abstract

Epithelioid sarcoma, though typically seen in young males, can occur in elderly patients and mimic benign lesions. Clinicians should maintain a high index of suspicion for chronic, slow‐growing extremity masses as accurate histopathological and immunohistochemical evaluation with complete surgical excision is crucial for optimal outcomes.

## 1 Introduction

Epithelioid sarcoma (ES) is an uncommon, aggressive soft‐tissue malignancy of mesenchymal origin, representing less than 1% of all soft‐tissue sarcomas [1]. Lack of comprehensive cancer registries and diagnostic facilities in Sub‐Saharan African countries hinders the accurate assessment of the true prevalence and incidence rates of soft‐tissue sarcoma including ES [2]. It most frequently affects young adult males and typically presents as a painless, slow‐growing nodular mass located in the dermis, subcutaneous tissue, or along tendinous and fascial planes [3]. Two main subtypes are recognized: distal (classical) and proximal ES, classified primarily by the anatomic location [4]. Distal‐type ES usually arises in the distal extremities and demonstrates epithelioid and spindled cells with central geographic necrosis, whereas proximal‐type ES occurs in deep soft tissues of the trunk or pelvis and shows marked pleomorphism, rhabdoid morphology, and signet‐ring–like vacuolation without the pseudo‐granulomatous pattern of distal lesions.

First described by Enzinger in 1970 after frequent misclassification as synovial sarcoma, ES remains diagnostically challenging due to its morphologic overlap with granulomatous disease, chronic inflammation, and epithelial neoplasms [3]. Accurate diagnosis requires integration of clinical features, anatomic site, histopathology, and immunohistochemistry, including evaluation for loss of INI1 (SMARCB1), a recently recognized diagnostic hallmark [5, 6, 7, 8, 9, 10].

We report an unusual case of distal‐type ES in a 70‐year‐old woman, underscoring the importance of considering this entity in atypical demographic settings.

## 2 Case History

A 70‐year‐old woman with a history of well‐controlled hypertension presented with a gradually enlarging swelling on the right forearm of 7 months' duration. The lesion began as a small papule and progressively increased in size, becoming mildly tender with overlying skin peeling but no ulceration, discharge, or functional limitation. Past medical history was notable for endometrioid endometrial carcinoma, for which the patient underwent total abdominal hysterectomy with bilateral salpingo‐oophorectomy, followed by adjuvant chemotherapy and radiotherapy.

On examination, she was alert (GCS 15/15), afebrile (36°C), with blood pressure 160/75 mmHg, pulse 85 bpm, and oxygen saturation 97% on room air. There was no pallor, jaundice, peripheral edema, or palpable regional lymphadenopathy. Local examination revealed a 2 × 3 cm firm, mobile swelling on the dorsal aspect of the right forearm with scaly, hyperpigmented overlying skin and mild tenderness.

## 3 Differential Diagnosis, Investigations and Treatment

Laboratory tests showed hemoglobin 11.8 g/dL, eGFR 103 mL/min, and serum urea 2.95 mmol/L. Ultrasonography demonstrated a vascularized, soft‐tissue mass extending to the subcutaneous tissue without involvement of deeper fascia; the mass had mixed echogenicity and ill‐defined borders. A clinical diagnosis of granulomatous lesion was entertained because the mass was superficial, slowly progressive, mildly tender, and associated with overlying skin changes, without ulceration or rapid growth—features that commonly overlap with benign inflammatory or granulomatous conditions in routine clinical practice.

**FIGURE 1:**
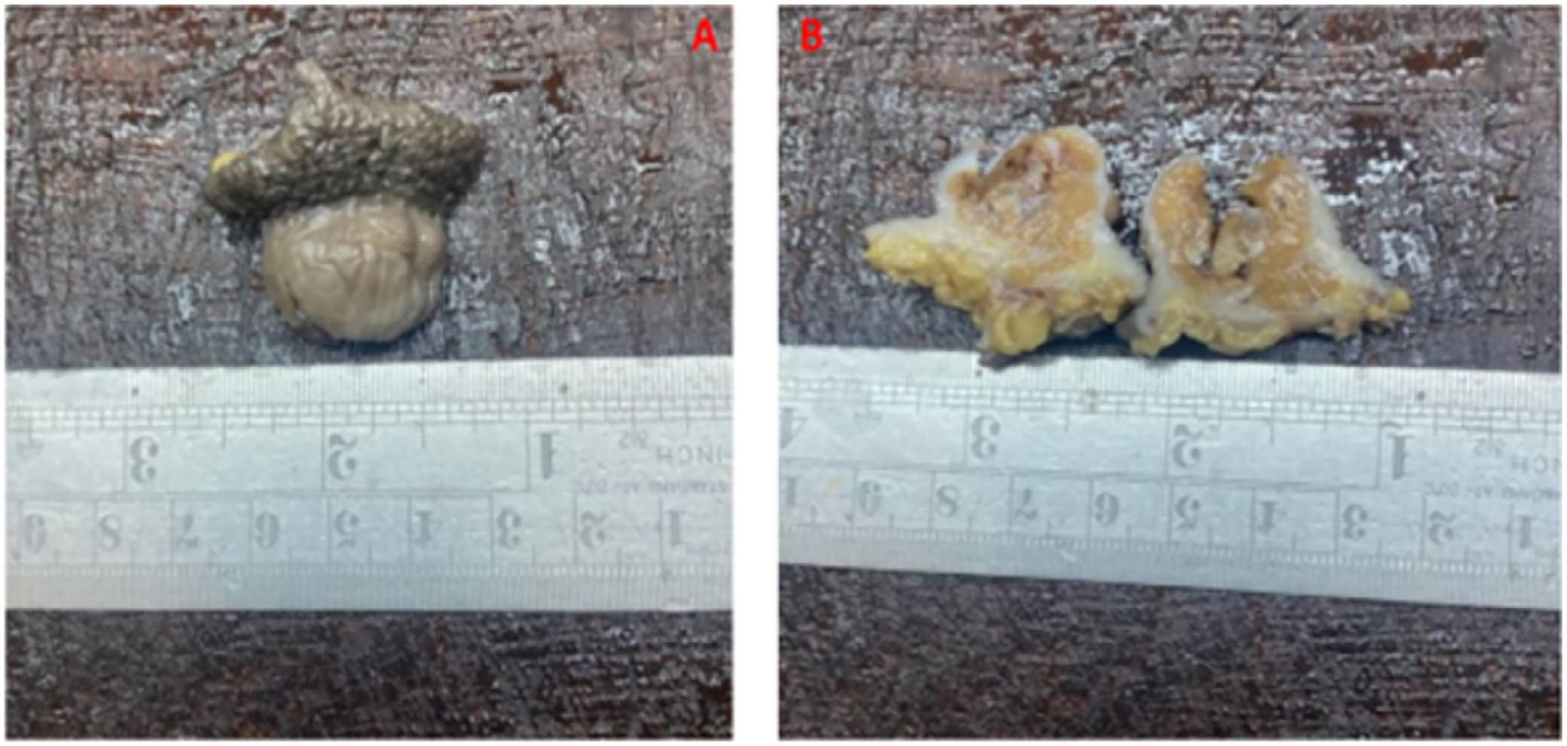
A wide local excisional biopsy demonstrates a nodular tumor with exophytic and endophytic growth patterns measuring 4 by 3 cm, partially covered by intact nodulated skin (A); cut surface is glistering tan to brown with firm consistency (B).

**FIGURE 2:**
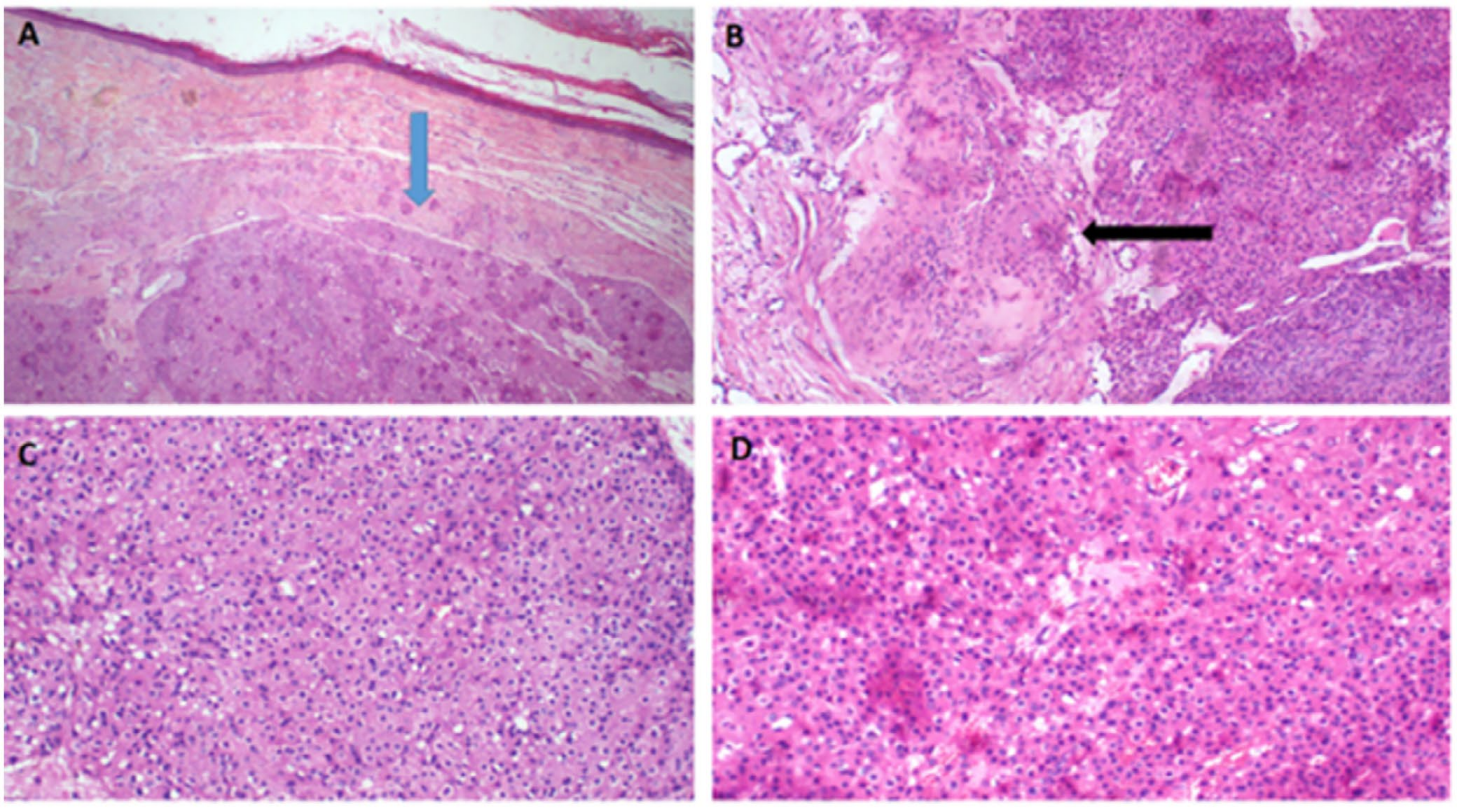
Histopathology of epithelioid sarcoma demonstrates a nodular lesion with infiltrative borders, centered in the deep dermis and subcutis (blue arrow), H&E staining at 2 x original magnification (A); the tumor simulates granulomatous process without necrosis (pseudogranulomatous morphology) (black arrow), H&E staining at 4 x original magnification (B); fairly uniform plump small‐to‐medium sized cells with eosinophilic cytoplasm, H&E staining at 10 x original magnification (C); mildly atypical nuclei with vesicular chromatin and small nucleoli, H&E staining at 20 x original magnification (D).

**FIGURE 3:**
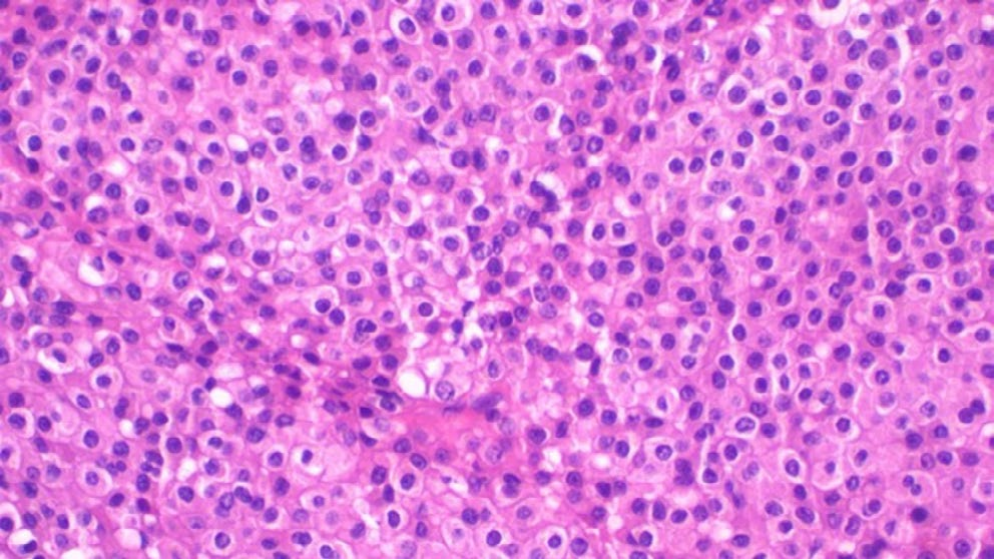
Photomicroscopy of epithelioid sarcoma displaying rhabdoid‐like features including sheets of epithelioid cells with glassy intracytoplasmic hyaline‐like inclusions and vesicular nuclei, H&E staining 40 × original magnification.

The patient underwent wide local excision with clear macroscopic margins. The excised specimen measured 4 × 3 cm and was partially covered by nodulated skin (Figure 1A). On the cut section, the mass was firm and tan–brown (Figure 1B). Histopathological examination revealed a nodular lesion with infiltrative borders, centered in the deep dermis and subcutis. Tumor cells are fairly uniform, plump, small to medium sized with eosinophilic cytoplasm. The cells have mildly atypical nuclei with vesicular chromatin and small nucleoli. The tumor simulates a granulomatous process without central necrosis (pseudo‐granulomatous morphology). Surgical margins were negative, but the tumor was close to the margins (Figures 2, 3).

**FIGURE 4:**
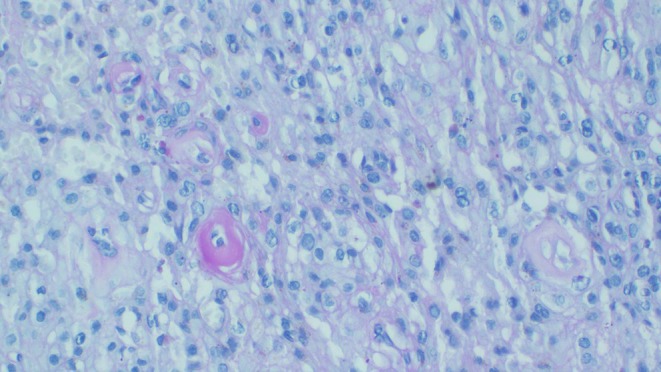
Periodic Acid–Schiff (PAS) of epithelioid sarcoma showing tumor cells with intracytoplasmic, bright magenta Periodic Acid–Schiff (PAS)‐positive inclusions with a perinuclear distribution, creating a halo‐like appearance around the nuclei at 10 x original magnification.

Immunohistochemistry showed strong positivity for vimentin, cytokeratin (AE1/AE3), and SMA. Additionally, the tumor cells demonstrated negativity for desmin and S100. The tumor had focal positivity of PAS stain (Figure 4). A diagnosis of ES with a differential diagnosis of glomus tumor was considered. Periodic acid–Schiff (PAS) staining demonstrated cytoplasmic PAS positivity within tumor cells, without the diffuse pericellular basement membrane staining characteristic of glomus tumor. These findings supported a diagnosis of classical (distal‐type) epithelioid sarcoma. Unfortunately, CD 34, as well as molecular tests including INI1/SMARCB1, were not performed as the tests are not available in our setting.

## 4 Conclusion and Results

The patient experienced an uneventful postoperative recovery with no immediate or delayed complications such as wound infection or functional impairment of the forearm. Histopathological margins were negative, and no evidence of lymph node involvement or distant metastasis was detected on postoperative imaging. The patient was thereby given radiotherapy 25 cycles. She has been enrolled in a structured surveillance program consisting of clinical examination and imaging at 3‐month intervals, and she remains free of local recurrence or distant disease at 6 months of follow‐up.

## 5 Discussion

Epithelioid sarcoma (ES) is a soft‐tissue malignancy, accounting for less than 1% of adult sarcomas with a peak incidence in the third to fourth decade of life. To date, only a few reports have been documented in the English literature [11]. Our case contrasts sharply with this demographic, occurring in a 70‐year‐old woman, underscoring the need to consider ES in atypical age groups and clinical settings. This case expands the age range in which distal‐type ES may present.

Two morphologic subtypes of ES are recognized. Distal (classical) ES usually presents as a superficial, slowly enlarging mass in the distal extremities and shows epithelioid and spindled cells with central necrosis, whereas proximal ES arises in deep soft tissues of the trunk or pelvis and exhibits marked pleomorphism and rhabdoid morphology [10]. The lesion in our patient's forearm, together with its long indolent course, is most consistent with the distal subtype despite the unusual age and sex.

Histopathological examination remains the cornerstone of diagnosis [3, 4]. ES is frequently misdiagnosed as benign or inflammatory lesions or as other malignancies such as synovial sarcoma, rhabdomyosarcoma, or poorly differentiated carcinoma [10]. Glomus tumor was considered in the differential diagnosis given the epithelioid morphology and focal SMA positivity. However, the absence of classic perivascular architecture, lack of diffuse strong SMA expression, cytokeratin positivity, and the PAS staining pattern argued against this diagnosis. Immunohistochemistry helps narrow the differential: ES is typically positive for vimentin, cytokeratins, EMA, and CD34 (in about 50% of cases), and loss of INI1 (SMARCB1) has emerged as a diagnostic hallmark [12]. In resource‐limited settings such as ours where molecular testing for INI1/SMARCB1 loss is unavailable, the diagnosis of ES relies heavily on clinical features, histomorphology, and a limited immunohistochemical panel [13]. This increases the risk of misdiagnosis, particularly in atypical presentations or when distinguishing ES from morphologic mimics such as synovial sarcoma, melanoma, or granulomatous lesions. Management may also be challenged by limited access to advanced imaging for staging, constrained surgical resources for achieving wide margins, and reduced availability of adjuvant radiotherapy or newer targeted agents. In such contexts, meticulous histopathologic evaluation, maximal use of available immunostains, complete surgical excision with clear margins, and structured long‐term surveillance become essential to optimize patient outcomes. In our case, pancytokeratin AE1/AE3 positivity as well as focal PAS stain positivity and desmin negativity supported the diagnosis of distal ES. INI1/SMARCB1 and CD 34 were not performed due to non‐availability of the test in our laboratory.

Surgical excision with wide margins remains the mainstay of treatment [11]. The index case underwent wide local excision with negative margins, though the tumor was in close proximity to the margins. Thereafter, the patient was given radiotherapy 25 cycles. According to ESMO guidelines, radiation therapy may be added before or after surgery to reduce local recurrence risk, especially if margins are close or positive [12]. ES has been shown to have a propensity for regional lymphatic spread, with reported lymph node metastasis rates ranging from 17% to 80% [13]. Given this risk of regional dissemination, sentinel lymph node biopsy may be a useful adjunct in the management of this tumor. Adverse prognostic factors in ES include large tumor size (> 5 cm), deep location, nuclear pleomorphism, high mitotic count, and presence of nerve or vascular invasion [14].

Despite optimal surgery, ES carries a high risk of local recurrence and distant metastasis, most commonly to the lungs, lymph nodes, bone, and brain. Vigilant long‐term follow‐up with serial clinical and imaging assessments is essential, and emerging targeted therapies such as EZH2 inhibitors may offer additional options in refractory disease [15, 16, 17]. ES can present in elderly patients as a superficial, indolent forearm lesion mimicking benign or inflammatory dermatoses and therefore warrants early biopsy and histopathological **evaluation**. Thus, this case emphasizes the importance for dermatologists to maintain a high index of suspicion for malignancy in persistent, slow‐growing nodules to pursue timely histopathological and immunohistochemical evaluation.

The lack of INI1/SMARCB1 and collagen IV immunostaining represents a limitation of this report. Diagnosing ES is difficult without INI1/SMARCB1 immunohistochemistry. In its absence, the tumor must be distinguished from several similar conditions, including granulomatous inflammation and synovial sarcoma, using mainly histologic features and a limited immunohistochemical panel (e.g., vimentin‐ and pancytokeratin‐positive and desmin‐negative). The lack of INI1 testing highlights the broader limitations in resource‐constrained settings, where advanced diagnostic tools are often unavailable, increasing the risk of delayed or incorrect diagnosis. Additionally, a 6‐month follow‐up period may be insufficient to fully evaluate recurrence or metastasis risk.

## 6 Conclusion

Epithelioid sarcoma is a rare mesenchymal tumor with aggressive behavior and poor prognosis. Early detection, radical surgical excision, and histopathological examination are important. Long‐term monitoring is essential for early detection of local recurrence and distant metastasis. This case highlights the importance of maintaining a broad differential diagnosis for chronic, slow‐growing extremity lesions in elderly patients and integrating clinical, histological, and immunohistochemical findings to achieve an accurate diagnosis.

## Author Contributions

**Furaha E. Kasyupa:** conceptualization, data curation, writing – original draft. **Jeremia J. Pyuza:** conceptualization, data curation, writing – review and editing. **Abitalis Mayengela:** data curation, writing – review and editing. **Agumbwike Mwakitwange:** writing – review and editing. **Deardiana Sengelela:** resources, writing – review and editing. **Patrick Amsi:** writing – review and editing. **Gilbert Nkya:** writing – review and editing. **Angela Pallangyo:** conceptualization, writing – review and editing. **Alex Mremi:** conceptualization, resources, supervision, writing – review and editing.

## Funding

This work did not receive funding from any source.

## Ethics Statement

The patient provided written informed consent to allow her de‐identified medical information to be used in this publication. A waiver for ethical approval was obtained from the authors' institution review board committee.

## Conflicts of Interest

The authors declare no conflicts of interest.

## Data Availability

The authors have nothing to report.
